# Primary isolated right ventricular failure after heart transplantation: prevalence, right ventricular characteristics, and outcomes

**DOI:** 10.1038/s41598-023-27482-x

**Published:** 2023-01-09

**Authors:** Peerapat Kaveevorayan, Nithi Tokavanich, Veraprapas Kittipibul, Thana Lertsuttimetta, Seri Singhatanadgige, Pat Ongcharit, Supanee Sinphurmsukskul, Aekarach Ariyachaipanich, Sarawut Siwamogsatham, Kanokwan Thammanatsakul, Supaporn Sritangsirikul, Sarinya Puwanant

**Affiliations:** 1 Division of Cardiovascular Medicine, Department of Medicine, Faculty of Medicine, Chulalongkorn University, Bangkok, Thailand; 2 Division of Cardiothoracic Surgery, Department of Surgery, Faculty of Medicine, Chulalongkorn University, Bangkok, Thailand; 3 Cardiac Center, King Chulalongkorn Memorial Hospital, Thai Red Cross Society, Bangkok, 10330 Thailand; 4 The Excellent Center of Organ Transplantation, King Chulalongkorn Memorial Hospital, Thai Red Cross Society, Bangkok, 10330 Thailand; 5 Faculty of Medicine, Chula Clinical Research Center, Chulalongkorn University, Bangkok, Thailand

**Keywords:** Cardiology, Cardiomyopathies, Heart failure

## Abstract

To determine the prevalence, right ventricular (RV) characteristics, and outcomes of primary isolated RV failure (PI-RVF) after heart transplant (HTX). PI-RVF was defined as (1) the need for mechanical circulatory support post-transplant, or (2) evidence of RVF post-transplant as measured by right atrial pressure (RAP) > 15 mmHg, cardiac index of < 2.0 L/min/m^2^ or inotrope support for < 72 h, pulmonary capillary wedge pressure < 18 mmHg, and transpulmonary gradient < 15 mmHg with pulmonary systolic pressure < 50 mmHg. PI-RVF can be diagnosed from the first 24–72 h after completion of heart transplantation. A total of 122 consecutive patients who underwent HTX were reviewed. Of these, 11 were excluded because of secondary causes of graft dysfunction (GD). PI-RVF was present in 65 of 111 patients (59%) and 31 (48%) met the criteria for PGD-RV. Severity of patients with PI-RVF included 41(37%) mild, 14 (13%) moderate, and 10 (9%) severe. The median onset of PI-RVF was 14 (0–49) h and RV recovery occurred 5 (3–14) days after HTX. *Severe* RV failure was a predictor of 30-day mortality (HR 13.2, 95% CI 1.6–124.5%, *p* < 0.001) and post-transplant dialysis (HR 6.9, 95% CI 2.0–257.4%, *p* = 0.001). Patients with *moderate* PI-RVF had a higher rate of 30-day mortality (14% vs. 0%, *p* = 0.014) and post-operative dialysis (21% vs. 2%, *p* = 0.016) than those with *mild* PI-RVF. Among patients with mild and moderate PI-RVF, patients who did not meet the criteria of PGD-RV had worsening BUN/creatinine than those who met the PGD-RV criteria (*p* < 0.05 for all). PI-RVF was common and can occur after 24 h post-HTX. The median RV recovery time was 5 (2–14) days after HTX. Severe PI-RVF was associated with increased rates of 30-day mortality and post-operative dialysis. Moderate PI-RVF was also associated with post-operative dialysis. A revised definition of PGD-RV may be needed since patients who had adverse outcomes did not meet the criteria of PGD-RV.

## Introduction

Primary graft dysfunction (PGD) is a leading cause of early mortality after heart transplantation^1,2^. Right ventricular PGD (PGD-RV) can cause devastating early post-transplant hemodynamic complications requiring mechanical circulatory support^1–5^. Primary isolated RV failure (PI-RVF) or PGD-RV is a condition where there is clinical and/or hemodynamic evidence of RVF in the absence of pulmonary hypertension, RV injury, and cardiac allograft rejection^1–5^. The pathogenesis of post-heart transplant RV failure is multifactorial and complex^2,4,6–11^. Although ischemia during organ preservation, a consequence of brain death and inotrope effect, and reperfusion injury leads to RV ischemic injury and RV failure, the established pathogenesis of this condition is yet to be determined^10,12,13^. Currently, PGD-RV is defined as right ventricular (RV) failure restricted to 24 h after surgery^1–5^. Invasive hemodynamic evidence of PGD-RV includes elevated right atrial pressure (> 15 mmHg), normal pulmonary capillary wedge pressure (PCWP) (< 15 mmHg), and low cardiac index (CI) (< 2 L/min/m^2^)^1–5^. Recently, Alam et al.^14^ proposed that the dose and duration of inotropic support can be used to stratify the severity of RV failure when CI ≥ 2.0 L/min/m^2^. This revised approach effectively expands the definition to include more patients with diagnostic criteria of PGD-RV. However, the limitation of this approach still exists in real world practice. First, the onset of the 24-h period is unclear as to whether it refers to the time of completion of the transplanted anastomosis or at completion of surgical chest closure. Second, delayed onset of PGD-RV beyond 24 h after surgery is not included. Third, patients with interventricular septal shift in response to RV dilatation may have mildly elevated PCWP (i.e., PCWP of 16–18 mmHg). Accordingly, the main modification defining PI-RVF included using a pulmonary capillary wedge pressure (PCWP) cut-off < 18 mmHg and expanding time to diagnose from the first 24–72 h after completion of heart transplantation. In this study, we sought to investigate: (1) the prevalence of PI-RVF (with and without diagnostic criteria of PGD-RV) after heart transplantation, (2) RV characteristics and recovery assessed by invasive mean right atrial pressure (RAP) in patients with PI-RVF, (3) the clinical gaps between PGD-RV and PI-RVF, and (4) clinical outcomes in patients with PI-RVF.

## Methods

### Study population

The study protocol was approved by the Chulalongkorn University Institutional Review Board with a waiver of written informed consent. All methods were carried out in accordance with the tenets of the Declaration of Helsinki and the ethical standard guidelines and regulations. We retrospectively reviewed consecutive patients undergoing heart transplantation from January 1, 2008, to October 31, 2021. We excluded patients with combined organ transplantation, continuation of mechanical circulatory support (MCS) from pre-transplant to post-transplant period, concomitant left ventricular PGD, and secondary graft dysfunction including acute rejection, pulmonary hypertension or pulmonary vascular disease, and intraoperative RV injury as a surgical complication. Pulmonary hypertension precluding from study eligibility was defined as pre-transplant pulmonary arterial systolic pressure > 55 mmHg, and/or transpulmonary gradient ≥ 16 mmHg, and/or pulmonary vascular resistance > 5 Wood units. Clinical laboratory data and cardiac investigation were abstracted by one investigator from the electronic hospital informative system and transplant database of the Excellent Center of Organ Transplantation (ECOT). Pre-transplant hemodynamics assessed by right heart catheterization closest to the transplant dates were reviewed.

### Definitions

All definitions were based on the International Society for Heart and Lung Transplantation (ISHLT) consensus conference report^4^ and were modified from the proposed revised diagnostic criteria of PGD-RV proposed by Alam et al.^14^ (Table 1A). The main modification defining PI-RVF included using a pulmonary capillary wedge pressure (PCWP) cut-off < 18 mmHg, elimination of the criteria for cardiac index (CI) < 2 L/min/m^2^ in the presence of multiple inotropic support, and expanding time to diagnose from the first 24–72 h after completion of heart transplantation (Table 1B). *PI-RVF* was diagnosed in the first 72 h after completion of the cardiac transplant surgery and was defined as hemodynamic compromise requiring mechanical circulatory support OR hemodynamic evidence followed the criteria: (1) mean RAP > 15 mmHg, (2) pulmonary PCWP < 18 mmHg, (3) CI < 2 L/min/m^2^ or use of inotropic support, and (4) transpulmonary gradient (TPG) < 15 mmHg and/or pulmonary artery systolic pressure < 50 mmHg.

**Table 1:** (A) Current modified diagnostic criteria of primary graft dysfunction -right ventricle (PGD-RV)^14^. (B) Proposed diagnostic criteria of primary isolated right ventricular failure (PI-RVF).

	(A) Onset	(B) Hemodynamic or clinical criteria
**(A)**		
PGD-RV Mild	Within 24 h after surgery	All of the following (3 of 3): (1) RAP > 15 mmHg (2) PCWP < 15 mmHg (3) CI < 2.0 L/min/m^2^ or off inotrope or requiring low dose* inotropes for < 72 h post-transplant **and** (1) TPG < 15 mmHg and/or (2) PASP < 50 mmHg
PGD-RV Moderate	Within 24 h after surgery	All of the following (3 of 3): (1) RAP > 15 mmHg (2) PCWP < 15 mmHg (3) CI < 2.0 L/min/m^2^ or escalating of inotrope requirements or instability to wean inotropes > 72 h post-transplant **and** (1) TPG < 15 mmHg and/or (2) PASP < 50 mmHg
PGD-RV Severe	Within 24 h after surgery	The need for RVAD
**(B)**		
*PI-RVF Mild*	**24–72 h** after surgery	All the following (3 of 3): (1) RAP > 15 mmHg (2) PCWP < **18 mmHg** (3) CI < 2.0 L/min/m^2^ or off inotrope or requiring low dose* inotropes for < 72 h post-transplant **and** **(1) not bold (1)**TPG < 15 mmHg and/or (2) PASP < 50 mmHg
*PI-RVF Moderate*	**24–72** h (bold h) after surgery	All the following (3 of 3): (1) RAP > 15 mmHg (2) PCWP < **18 mmHg** (3) CI < 2.0 L/min/m^2^ or escalating of inotrope requirements or instability to wean inotropes > 72 h post-transplant **and** **not bold (1)**not boldTPG < 15 mmHg and/or (2) PASP < 50 mmHg
*PI-RVF Severe*	**24–72** h (bold h)after surgery	The need for RVAD or ECMO

*CI* cardiac index, *PASP* pulmonary systolic pressure, *PCWP* pulmonary capillary wedge pressure, *PGD-RV* primary graft dysfunction- right ventricle, *RVAD* right ventricular assisted device, *RAP* right atrial pressure, *TPG* transpulmonary gradient.

*Low dose was defined by the following criteria: (1) single inotrope infusion: dobutamine < 7.5 µg (mcg)/kilogram (kg)/minute (min) or milrinone < 0.5 mcg/kg/min or epinephrine < 0.02 mcg/kg/min, or (2) two inotropic infusion: dobutamine < 3 mcg/kg/min or milrinone < 0.25 mcg/kg/min or epinephrine < 0.01 mcg/kg/min.^14^.

*“Mild” PI-RVF* was defined as: (1) mean RAP > 15 mmHg, (2) PCWP < 18 mmHg, (3) CI < 2 L/min/m^2^ or the condition of no inotropes or requiring low dose inotropes for < 72 h post-transplant, and (4) transpulmonary gradient (TPG) < 15 mmHg and pulmonary artery systolic pressure < 50 mmHg. Low dose was defined by the following criteria: (1) single inotrope infusion: dobutamine < 7.5 µg (mcg)/kilogram (kg)/minute (min) or milrinone < 0.5 mcg/kg/min or epinephrine < 0.02 mcg/kg/min, or (2) two inotropic infusion: dobutamine < 3 mcg/kg/min or milrinone < 0.25 mcg/kg/min or epinephrine < 0.01 mcg/kg/min^14^.

*“Moderate” PI-RVF* was defined as: (1) mean RAP > 15 mmHg, (2) PCWP < 18 mmHg, (3) CI < 2 L/min/m^2^ or condition requiring escalating inotropic doses or inability to wean inotropes > 72 h post-transplant^14^, and (4) transpulmonary gradient (TPG) < 15 mmHg and/or pulmonary artery systolic pressure < 50 mmHg.

*“Severe” PI-RVF* was defined as a need for MCS including extracorporeal membrane oxygenation (ECMO) or use of ventricular assisted device (VAD)^4,14^.

The *onset* of RV failure was determined by the time from the completion of surgical sternal closure to the onset of RAP > 15 mmHg. The *peak* of RV failure was determined by the time from completion of surgical sternal closure to the time of the highest RAP (> 15 mmHg). The *recovery* of RV failure was determined by the time from the completion of surgical sternal closure to the onset of RAP < 15 mmHg in patients who developed primary isolated RV failure. The *duration* of RV failure was determined by the time duration between the onset of RV failure and recovery from RV failure. The onset of RV failure was sub-classified to pattern A (onset of RV failure exhibited within 24 h after completion of sternal closure), pattern B (onset of RV failure exhibited between 24 and 48 h after completion of sternal closure), or pattern C (onset of RV failure exhibited after 48 h after completion of sternal closure).

### Donor procurement and post-transplant protocol

Donor hearts were procured after brain death declaration. Heart procurement was followed by the standard protocol using cold storage (4 °C) with preservation solution (Custodial, Newtown, PA, USA) of 2000–3000 ml. All heart transplant surgical techniques were biatrial anastomoses. Before January 2014, rabbit anti-thymocyte globulin was used for induction therapy. In February 2014, low dose basiliximab was universally implemented as the institutional protocol^15^. The adjunctive immunosuppression was comprised of oral mycophenolate sodium (MPS) 720 mg 6 h prior to the operation and two 500 mg doses of intravenous methylprednisolone intraoperatively. All patients received cyclosporine or tacrolimus, MPS, and steroids. Delayed calcineurin inhibitor (CNI) initiation after post-operative day 3 was universally implemented in all patients who received induction therapy since we found a very low incidence of > 1R ACR in the first 4 weeks post-transplant in our center. CNI-free or low-dose CNI regimen or delayed CNI initiation after 7 days post-transplant was commenced when the serum creatinine > 1.8–2 mg/dl. Post-transplant hemodynamic assessment was performed using a pulmonary artery (PA) catheter and an arterial line placed in the operating room prior to the transplant surgery in all patients. A chest radiograph combined with a good quality pressure waveform of pulmonary arterial pressure and PCWP was used to verify the position of the PA catheter tip in the intensive care unit (ICU). According to hemodynamic measurement protocols for the operating room and ICU, pressure transducers were calibrated to the zero level at the midaxillary line in the supine position before taking measurements. Mean right atrial pressure, pulmonary arterial pressure, and arterial pressure were measured and recorded every hour or more frequently if clinically indicated. PCWPs, cardiac output, CI, and systemic vascular resistance (SVR) were measured and recorded every 4 h post-operation or more frequently if clinically indicated. After 96 h post-transplant, the frequencies of those invasive hemodynamic measurements were judged on a case-by-case basis by the attending physicians. Epinephrine, milrinone, and dobutamine were the most frequently used inotropes during post-operative period. Nitric oxide or inhaled iloprost was given for RV failure in selected cases.

The maintenance immunosuppressive agents followed the standard protocol. Cyclosporine was administered orally 2–5 mg/kg/d to achieve blood trough levels of 250–275 ng/mL during week 4, 200–250 ng/mL during week 4 to month 6, and 150–200 ng/mL during month 6–12. In February 2014, tacrolimus was administered orally 0.05–0.1 mg/kg/d in place of cyclosporine to achieve blood trough levels of 10–14 ng/mL during week 4, 10–12 ng/mL during week 4 to month 6, and 10 ng/mL during month 6–12. MPS was administered orally 48 h post-transplant to maintain the dose of 1–1.5 g twice a day. Oral prednisolone was initiated at 1 mg/kg/d and then tapered to 0.5 mg/kg/d by day 7, 0.35 mg/ kg/d by week 3, and 0.1 mg/kg/d until stopping after month 6. Prophylaxis for infection included trimethoprim-sulfamethoxazole and fluconazole. A pre-emptive strategy for cytomegalovirus (CMV) treatment was applied to all patients.

Endomyocardial biopsies were obtained at numerous times post-transplant (Weeks 1, 2, 3, 4, 6, 8, 12, 14, 16, 20, 24, 32, 40, and 48 and every year for 5 years).

### Outcomes

Death was determined by the ECOT database, medical records, death certificates, or telephone reviews. Renal replacement therapy (RRT) or dialysis was performed by short-term continuous veno-venous hemofiltration in the ICU during the post-operative period. BUN and creatinine on post-operative day 3 represented post-operative blood urea nitrogen (BUN) and creatinine (the peak/worsening RV failure occurred on post-operative day 3). Graft loss was defined as a clinical syndrome of heart failure with objective evidence of cardiac structural abnormalities assessed by echocardiogram including left or right ventricular systolic dysfunction or elevated filling pressures. Acute cellular rejection (ACR) was defined using the revised criteria. Histopathological examination for ACR was performed in accordance with the ISHLT 2005 working formulation: (i) Grade 0R: no rejection; (ii) Grade 1R: an infiltrate with only one focus of myocyte damage or none; (iii) Grade 2R: an infiltrate with multifocal myocyte damage; and (iv) Grade 3R: diffuse myocyte damage with or without edema, hemorrhage, or vasculitis^16^.

### Statistical analysis

Frequency, percentage, median, and mean ± standard deviation (SD) were employed to describe the data. The mean difference between variables were compared using a student’s t-test for variables with normal distribution, a Wilcoxon–rank sum test for variables with non-normal distribution, and Analysis of Variance (ANOVA) for variables with more than 2 groups. Categorical variables were compared using a Chi squared test or Fischer’s exact test, where appropriate. Univariate and multivariate analyses were employed to identify the pre-operative predictors of primary isolated RV failure. Adjustment factors used in the multivariate model included age, pre-operative BUN > 43 mg/dL, direct bilirubin > 1.5 mg/dl, pre-transplant PVR (Wood units), donor age, ischemic time, and induction therapy. Due to the small number of deaths in 30-day mortality and subjects with post-operative dialysis, a multivariate analysis was not performed. Kaplan–Meier curves were constructed to estimate survival or event-free survival between groups. Significant differences in survival or events were based on the log-rank test. *P* values < 0.05 were considered significant. The data were analyzed using software IBM® SPSS® statistics version 28.0.0.0 and JMP statistical software.

## Results

From January 2008 to October 2021, 122 consecutive patients undergoing heart transplantation were reviewed for study inclusion. Eleven were excluded (3 acute rejection, 3 concomitant left ventricular PGD, 2 continuation of extracorporeal membrane oxygenation support from pre-transplant period, 1 pulmonary hypertension, 1 heart-kidney transplantation, and 1 intraoperative RV injury). Of the 3 patients with early rejections, one patient who had hyperacute rejection presumably due to non-HLA antigen (negative pre-transplant panel reactive antibody and retrospective crossmatch) developed it immediately in the operating room, one had grade 2R acute cellular rejection which developed at pos-operative day 7, and one had antibody-mediated rejection that developed at day 16 post-transplant. A total of 111 patients were enrolled in the study. Baseline characteristics of patients are shown in Table 2. The mean age of patients was 41 ± 16 years; 68 (72%) were male. INTERMACS profile 1–3 and pre-transplant MCS were present in 23% and 7% of patients, respectively.

**Table 2:** Baseline characteristics.

	Total (n = 111)	No or mild isolated RV failure (n = 87)	Moderate isolated RV failure (n = 14)	Severe isolated RV failure (n = 10)	*P* value
**Recipients**					
Age (years)	41 ± 16	40 ± 16	47 ± 13	44 ± 18	0.284
Female (n, %)	32 (28%)	26 (30%)	1 (7%)	5 (50%)	0.060
BMI (kg/m^2^)	20.2 ± 3.5	20.2 ± 3.6	20.2 ± 2.8	20.0 ± 3.3	0.981
Congenital heart disease (n, %)	4 (4%)	4 (5%)	0 (0%)	0 (0%)	1.000
BUN (mg/dL)	28.5 ± 15.8	26.5 ± 15.0	30.4 ± 14.1	43.3 ± 18.3	0.005*
Creatinine (mg/dL)	1.3 ± 1.1	1.3 ± 1.3	1.5 ± 0.4	1.4 ± 0.4	0.886
Direct bilirubin (mg/dL)	1.2 ± 1.2	1.0 ± 1.0	1.0 ± 0.6	2.8 ± 1.9	< 0.001*
Diabetes (n, %)	12 (11%)	10 (12%)	2 (14%)	0 (0%)	0.642
INTERMACS profile 1–3 (n, %)	26 (23%)	22 (25%)	2 (14%)	2 (20%)	0.784
Inotrope dependence (n, %)	21 (19%)	17 (20%)	1 (7%)	3 (30%)	0.373
Pre-operative MCS (n, %)	8 (7%)	8 (9%)	0 (0%)	0 (0%)	0.651
ECMO	7 (6%)				
Bi-VAD	1 (1%)				
Hemoglobin < 10 g/dl (n, %)	11 (10%)	9 (10%)	1 (7%)	1 (10%)	1.000
Direct bilirubin > 1.5 mg/dl (n, %)	26 (24%)	16 (19%)	2 (14%)	8 (80%)	< 0.001*
BUN > 43 mg/dl (n, %)	19 (17%)	10 (12%)	3 (21%)	6 (60%)	0.001*
Creatinine > 1.8 mg/dl (n, %)	15 (14%)	10 (12%)	2 (14%)	3 (30%)	0.200
Pre-transplant RAP (mmHg)	10.8 ± 6.0	10.5 ± 5.6	9.7 ± 7.2	14.8 ± 7.0	0.077
Pre-transplant TPG (mmHg)	9.3 ± 4.8	9.6 ± 5.0	8.0 ± 3.5	8.9 ± 4.0	0.516
Pre-transplant PVR (Wood units)	3.1 ± 2.0	3.2 ± 2.2	3.0 ± 1.4	3.2 ± 2.0	0.947
Pre-transplant PVR > 3 Wood units (n, %)	44 (47%)	32 (45%)	7 (50%)	5 (56%)	0.828
Pre-transplant TPG > 12 mmHg (n, %)	21 (21%)	18 (24%)	0 (0%)	3 (30%)	0.065
Pre-transplant RAP > 12 mmHg (n, %)	37 (33%)	29 (33%)	2 (14%)	6 (60%)	0.079
Pre-transplant PA SaO_2_ (%)	64.3 ± 12.7	65.0 ± 13.6	59.0 ± 8.0	65.3 ± 7.6	0.504
**Donor**					
Age (years)	28 ± 9	27 ± 9	30 ± 9	30.0 ± 8	0.361
Female (n, %)	13 (12%)	12 (13%)	0 (0%)	1 (10%)	0.365
BMI (kg/m^2^)	23.0 ± 3.0	22.9 ± 3.0	24.1 ± 3.0	23.0 ± 2.7	0.336
Inotrope score^‡^	19.8 ± 17.9	19.7 ± 16.3	27.3 ± 7.3	15.5 ± 14.3	0.580
History of chest compression (n, %)	3 (2.7%)	2 (2.3%)	1 (7.1%)	0 (0%)	0.521
Donor intracranial hemorrhage (n, %)	20 (18%)	14 (16%)	3 (21.4%)	3 (30%)	0.405
**Procedural characteristics**					
RADIAL score^¶^	1.7 ± 1.0	1.7 ± 1.0	1.5 ± 0.9	1.9 ± 1.0	0.647
RADIAL score > 2 (n, %)	22 (19.8%)	18 (20.7%)	2 (14.3%)	2 (20%)	0.913
Female donor to male recipient (n, %)	6 (5%)	6 (7%)	0 (0%)	0 (0%)	0.766
Undersized donor (n, %)	29 (26%)	23 (26%)	3 (21%)	3 (30%)	0.932
Crossed ABO blood group (n, %)	29 (26%)	21 (24%)	6 (43%)	2 (20%)	0.339
Ischemic time > 240 min (n, %)	56 (51%)	45 (52%)	7 (50%)	4 (40%)	0.781
Ischemic time (min)	226 ± 63	229 ± 7	208 ± 17	224 ± 20	0.528
**Post-operative medical therapy**					
Nitric oxide use (%)	30 (27%)	14 (16%)	6 (43%)	10 (100%)	< 0.001
Nitric oxide (ppm)**	38 ± 19	30 ± 5	44 ± 8	47 ± 6	0.060
Levosimadan (% use)	16 (1.4%)	10 (12%)	3 (21%)	3 (30%)	0.209
Epinephrine (% use)	74 (67%)	52 (60%)	13 (93%)	9 (90%)	< 0.001
Norepinephrine (% use)	8 (7%)	1 (1%)	3 (21%)	4 (40%)	< 0.001
Dobutamine (% use)	64 (58%)	46(53%)	9 (64%)	9 (90%)	0.045
Dopamine (% use)	44 (40%)	30 (35%)	9 (64%)	5 (50%)	0.087
Milrinone (% use)	72 (66%)	53 (61%)	12 (86%)	7 (70%)	0.150
Isoproterenol (% use)	53 (49%)	40 (48%)	7 (50%)	6 (60%)	0.758

*Bi-VAD* biventricular assisted device, *BMI* body mass index, *BUN* blood urea nitrogen, *INTERMACS* Interagency Registry for Mechanically Assisted Circulatory Support, *MCS* mechanical circulatory support, *PA* pulmonary artery, *PVR* pulmonary vascular resistance, *RAP* right atrial pressure, *RV* right ventricular, *SaO2* oxygen saturation, *TPG* transpulmonary pressure gradient.

*P value < 0.05 is statistical significance.

^‡^Inotropic score = dopamine (× 1) + dobutamine (× 1) + milrinone (× 15) + epinephrine (× 100) + norepinephrine (× 100) with each drug dose in microgram/kilogram/minute.

^¶^RADIAL score = Right atrial pressure ≥ 10 mmHg, recipient Age ≥ 60 years, Diabetes mellitus, Inotrope dependence, donor Age ≥ 30 years, Length of ischemic time ≥ 240 min.

**Post-operative nitric oxide doses were calculated by mean doses in ppm during post-operative day 0–3.

### The prevalence of post-transplant primary isolated RV failure

**Figure 1:**
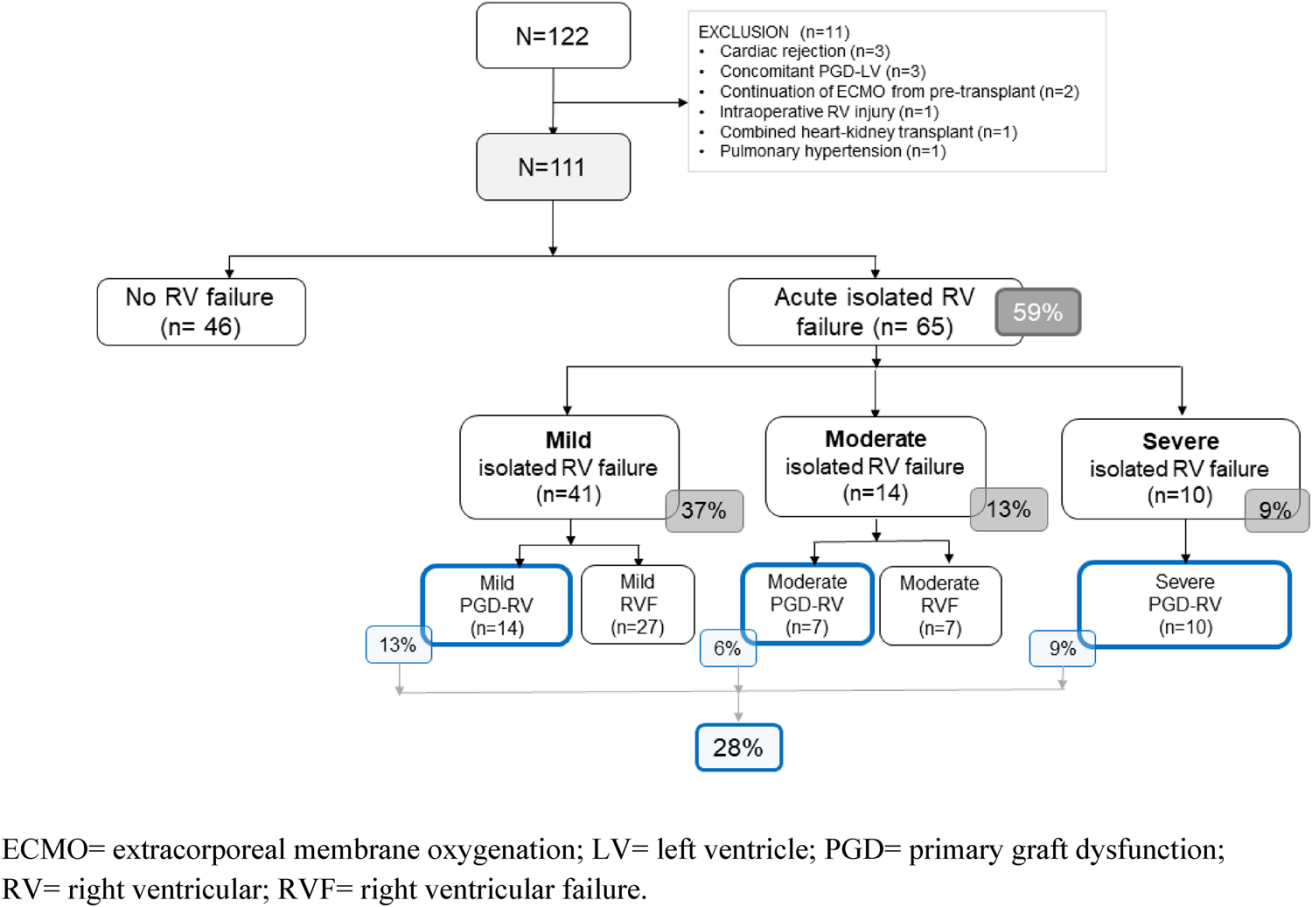
Categories and subcategories of study patients.

Of 111 study patients, 65 (59%) had post-transplant PI-RVF (Fig. 1). Of these, 41 (37%), 14 (13%) and 10 (9%) had mild, moderate, and severe PI-RVF, respectively. Baseline characteristics in no/mild, moderate, and severe PI-RVF after heart transplantation are shown in Table 2. Serum BUN and bilirubin increased with the scale severity of RV failure.

### Characteristics of post-transplant primary isolated RV failure

#### Onset of RV failure

**Figure 2:**
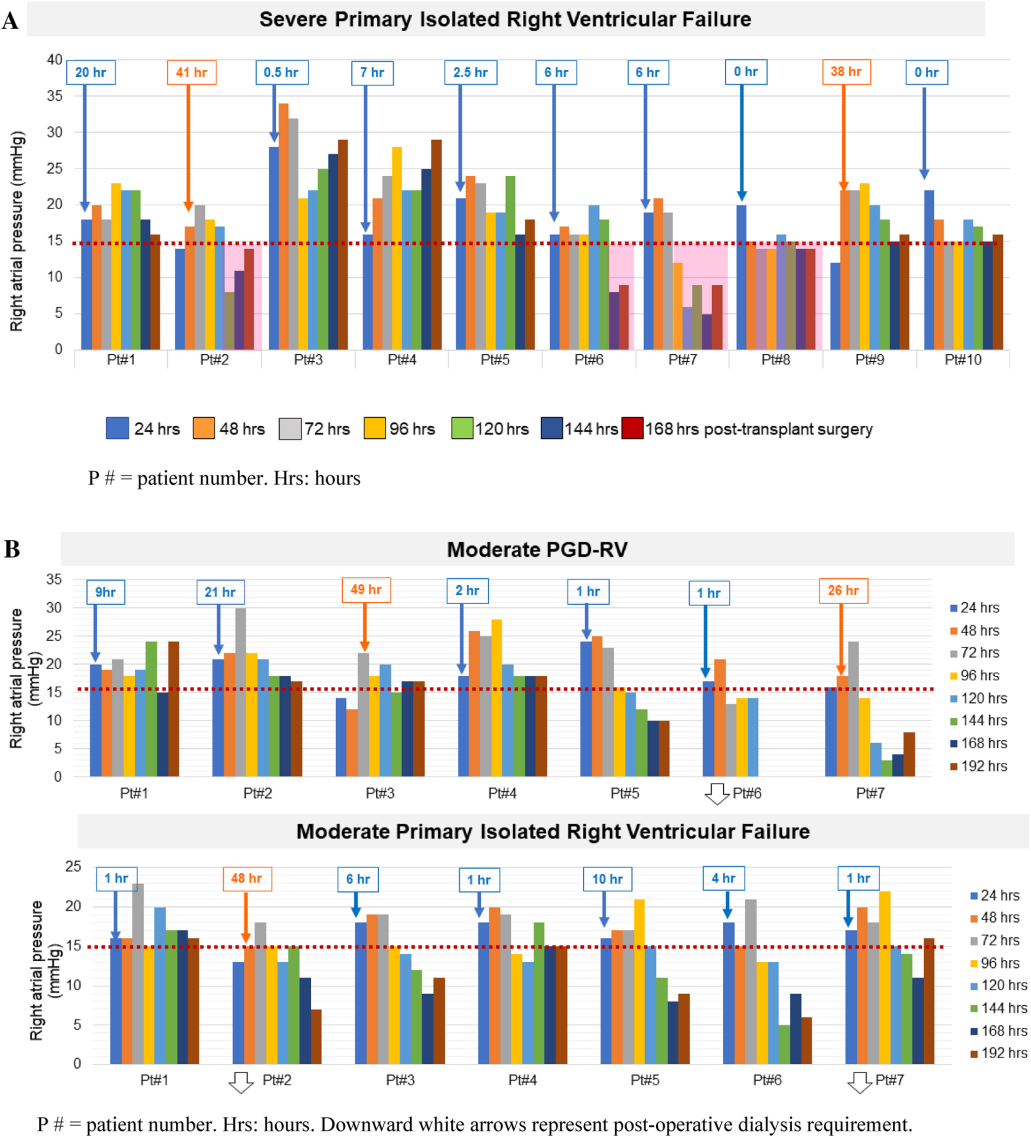
(**A**) Distribution of post-operative mean right atrial pressures in patients with severe right ventricular failure. (**B**) Distribution of post-operative mean right atrial  pressures in patients with moderate right ventricular failure.

Median time to the onset of PI-RVF was **14 (0–49) h **after completion of cardiac transplant surgery. Among the 65 patients with post-transplant PI-RVF, 36 (55%), 23 (35%) and 6 (9%) developed the onset of RV failure within 24 h (pattern A), between 24 and 48 h (pattern B) and after 48 h (pattern C) of completion of cardiac transplant surgery, respectively. In the subgroup of *severe* PI-RVF, 2 of 10 (14%) had a time to the onset of RV failure after 24 h (38 and 41 h) after completion of heart transplant surgery. In the subgroup of *moderate* PI-RVF, 3 patient (21%) had an onset to RV failure after 24 h after completion of heart transplant surgery. Figure 2A,B show distributions of mean RAP in patients with moderate and severe PI-RVF.

#### Peak of RV failure

The peak of elevated RAP was observed on post-operative **day 3 (2–4).**

#### Recovery of RV failure

Among the patients with post-transplant PI-RVF, RV recovery was observed on post-operative **day 5 (3–14).** Resolution of RV failure within 7 days post cardiac transplantation occurred in 49 patients (75%). In the subgroup of *severe* PI-RVF, 4 of 10 patients (40%) had RV recovery within 7 days post-operation (Fig. 2A). In the subgroup of *moderate* post-operative RV failure, 7 (50%) had RV recovery within 7 days post-operation and 3 required post-operative dialysis (Fig. 2B). Table 3 shows the mean RAP in overall patients and stratified by the scale severity of RV failure.

**Table 3:** RV characteristics and post-transplant clinical outcomes.

	Total (n = 111)	No or mild isolated RV failure (n = 87)	Moderate isolated RV failure (n = 14)	Severe isolated RV failure (n = 10)	*P* value
**RV characteristics**					
Maximal RAP (mmHg)	25 ± 11	21 ± 2	27 ± 15	23 ± 5	0.596
Hours to onset of RAP > 15 mmHg (post-operative hours)	14 ± 21	23 ± 23	14 ± 25	12 ± 16	0.833
Days to onset of RAP > 15 mmHg (post-operative days)	1.6 ± 0.9	1.6 ± 0.9	1.5 ± 0.7	1.8 ± 1.1	0.658
Resolved RV failure (post-operative days)	5.7 ± 2.5	5.4 ± 2.4	6.0 ± 2.2	6.6 ± 3.5	0.360
**Post-transplant outcomes**					
Death (n, %)	34 (31%)	25 (29%)	3 (21%)	6 (60%)	0.121
30-day mortality (n, %)	12 (11%)	4 (5%)	2 (14%)	6 (60%)	< 0.001*
≥ 2R acute cellular rejection or significant antibody-mediated rejection (n, %)	0 (0%)	0 (0%)	0 (0%)	0 (0%)	
Graft loss (n, %)	0 (0%)	0 (0%)	0 (0%)	0 (0%)	
Post-operative dialysis in the absence of sepsis or CNI (n, %)	10 (10%)	1 (1%)	3 (21%)	6 (60%)	< 0.001*
Post-operative day 3 BUN (mg/dL)	50 ± 27	42 ± 20	63 ± 20	95 ± 40	< 0.001*
Post-operative day 3 creatinine (mg/dL)	2.0 ± 1.2	1.7 ± 1.0	2.5 ± 1.3	3.3 ± 1.4	< 0.001*
Length of stay (days)	44.9 ± 36.8	46.1 ± 39.4	45.4 ± 27.6	33.8 ± 21.5	0.609

*BUN* blood urea nitrogen, *CNI* calcineurin inhibitor, *RAP* right atrial pressure, *RV* right ventricular.

*P value < 0.05 is statistical significance.

#### Duration of RV failure

Among all patients, the median duration of RV failure was **4 (1–14) days.** In the subgroup of 10 patients with post-operative MCS support (1 short-term RVAD and 9 ECMO), 2 died on MCS. Of the 8 survivors of MCS support, median duration of MCS support was **6 (3–12) days.**

### Patients with post-transplant primary isolated RV failure who did NOT meet the PGD-RV criteria

Of 65 patients with post-transplant PI-RVF, 34 (52%) did not meet the criteria of PGD-RV because onset of RV failure occurred after 24 h from completion of cardiac transplant surgery (n = 29), cardiac index > 2 L/min/m^2^ (n = 3), or PCWP of 16–17 mmHg (n = 2). Of these patients, 27 and 7 had mild and moderate post-operative RV failure, respectively. One patient died at 61 days post-transplant due to severe infection. Table 4 illustrates clinical outcomes in patients with mild and moderate PI-RVF who met and did not meet the PGD-RV criteria. Death, 30-day mortality, ACR ≥ 2R, graft loss, length of stay, and requirement of post-operative dialysis were similar between those who met and did not meet the PGD-RV criteria. Among the patients with *mild* RV failure, those who did not meet the criteria of PGD-RV had higher BUN and creatinine on post-operative day 3 than those with PGD-RV (53 ± 22 vs. 32 ± 11 mg/dl, *p* < 0.01 for BUN, and 1.8 ± 0.8 vs 1.4 ± 0.4 mg/dl, *p* = 0.03 for creatinine). Among the patients with *moderate* RV failure, those who did not meet the criteria of PGD-RV had similar BUN and creatinine on post-operative day 3 than those with PGD-RV (71 ± 23 vs. 55 ± 14 mg/dl, *p* = 0.08 for BUN, and 2.9 ± 1.8 vs 2.2 ± 0.5 mg/dl, *p* = 0.56 for creatinine).

**Table 4:** Post-transplant clinical outcomes.

	No RV failure (n = 46)	Mild primary isolated RV failure		Moderate primary isolated RV failure		*P* value
		PGD-RV + (n = 14)	PGD-RV- (n = 27)	PGD-RV + (n = 7)	PGD-RV- (n = 7)	
**Post-transplant outcomes**						
Death (n, %)	14 (30%)	4 (29%)	7 (26%)	3 (43%)	0 (0%)	0.456
30 days mortality (n, %)	4 (9%)	0 (0%)	0 (0%)	2 (29%)	0 (0%)	0.076
≥ 2R acute cellular rejection or significant antibody-mediated rejection (n, %)	0 (0%)	0 (0%)	0 (0%)	0 (0%)	0 (0%)	
Graft loss (n, %)	0 (0%)	0 (0%)	0 (0%)	0 (0%)	0 (0%)	
Length of stay (days)	39 ± 29	41 ± 33	62 ± 53	34 ± 28	55 ± 25	0.102
Post-operative dialysis in the absence of sepsis or CNI (n, %)	0 (0%)	1 (7%)	0 (0%)	1 (14%)	2 (29%)	0.078
Post-operative day 3 BUN (mg/dL)	38 ± 19	32 ± 11	53 ± 22^‡^	55 ± 14	71 ± 23^¶^	< 0.001*
Post-operative day 3 creatinine (mg/dL)	1.7 ± 1.3	1.4 ± 0.4	1.8 ± 0.8^‡^	2.2 ± 0.5	2.9 ± 1.8	0.057

*BUN* blood urea nitrogen, *CNI* calcineurin inhibitor, *RAP* right atrial pressure, *RV* right ventricular.

PGD-RV + : patients who met the criteria of PGD-RV; PGD-RV-: patients who did not meet the criteria of PGD-RV.

^‡^*P* value < 0.05, compared between patients with mild PGD-RV and mild isolated RV failure with no PGD.

^¶^*P* value = 0.08, compared between patients with moderate PGD-RV and moderate isolated RV failure with no PGD.

*P ANOVA < 0.05.

### Outcomes of post-transplant acute isolated RV failure

**Figure 3:**
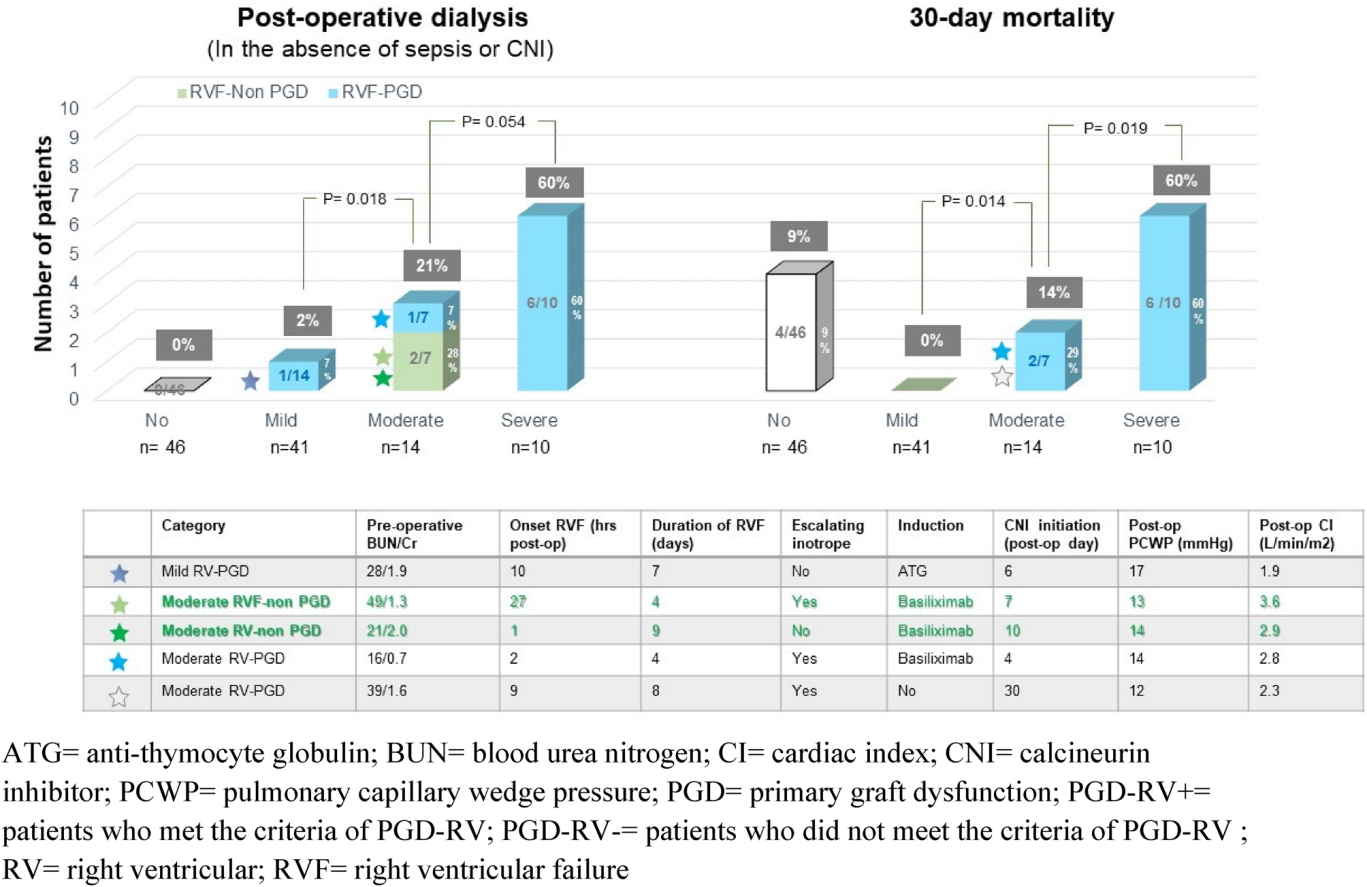
Post-operative requirement of dialysis and 30-day mortality in patients with mild and moderate right ventricular (RV) failure stratified by primary graft dysfunction (PGD) and non PGD and severe RV failure/PGD.

The follow-up outcomes were completed in 100% of patients. Over a median follow-up of 181 (2–2,314) days, there were 34 deaths (31%). At 30 days post transplantation, there was 12 deaths (11%). The causes of 30-day deaths included 3 treated infections, 1 limb ischemia associated with ECMO, 1 severe lung injury as a surgical complication, and 7 RV failures. The 30-day mortality rates for post-transplant severe, moderate, and no/mild PI-RVF were 60%, 14%, and 5%, respectively (*p* = 0.019 for severe vs. moderate RV failure, *p* = 0.014 for moderate vs. mild RV failure) (Table 3 and Fig. 3). Four deaths (3 due to infection and 1 due to lung injury) occurred in patients with *no* RV failure.

Among all patients, 13 patients required post-operative dialysis. Of these, 10 required initiation of dialysis in the absence of sepsis and before CNI initiation. The incidences of dialysis in the absence of sepsis and before CNI initiation for severe, moderate, and mild acute isolated RV failure were 60%, 21%, and 2%, respectively (*p* = 0.054 for severe vs. moderate RV failure, *p* = 0.018 for moderate vs. mild RV failure) (Table 3 and Fig. 3). Post-operative day 3 BUN and creatinine in patients with *severe* isolated RV failure was higher than those with *moderate* isolated RV failure (95 ± 40 vs. 63 ± 20 mg/dl, 0.02 for BUN, and 3.3 ± 1.4 vs. 2.5 ± 1.3, *p* < 0.01 for creatinine) (Table 3). Post-operative day 3 BUN and creatinine in patients with *moderate* isolated RV failure was higher than those with *mild* isolated RV failure (63 ± 20 vs. 42 ± 20 mg/dl, *p* = 0.01 for BUN, and 2.5 ± 1.3 vs 1.7 ± 1.0 mg/dl, *p* < 0.01 for creatinine) (Table 3).

**Figure 4:**
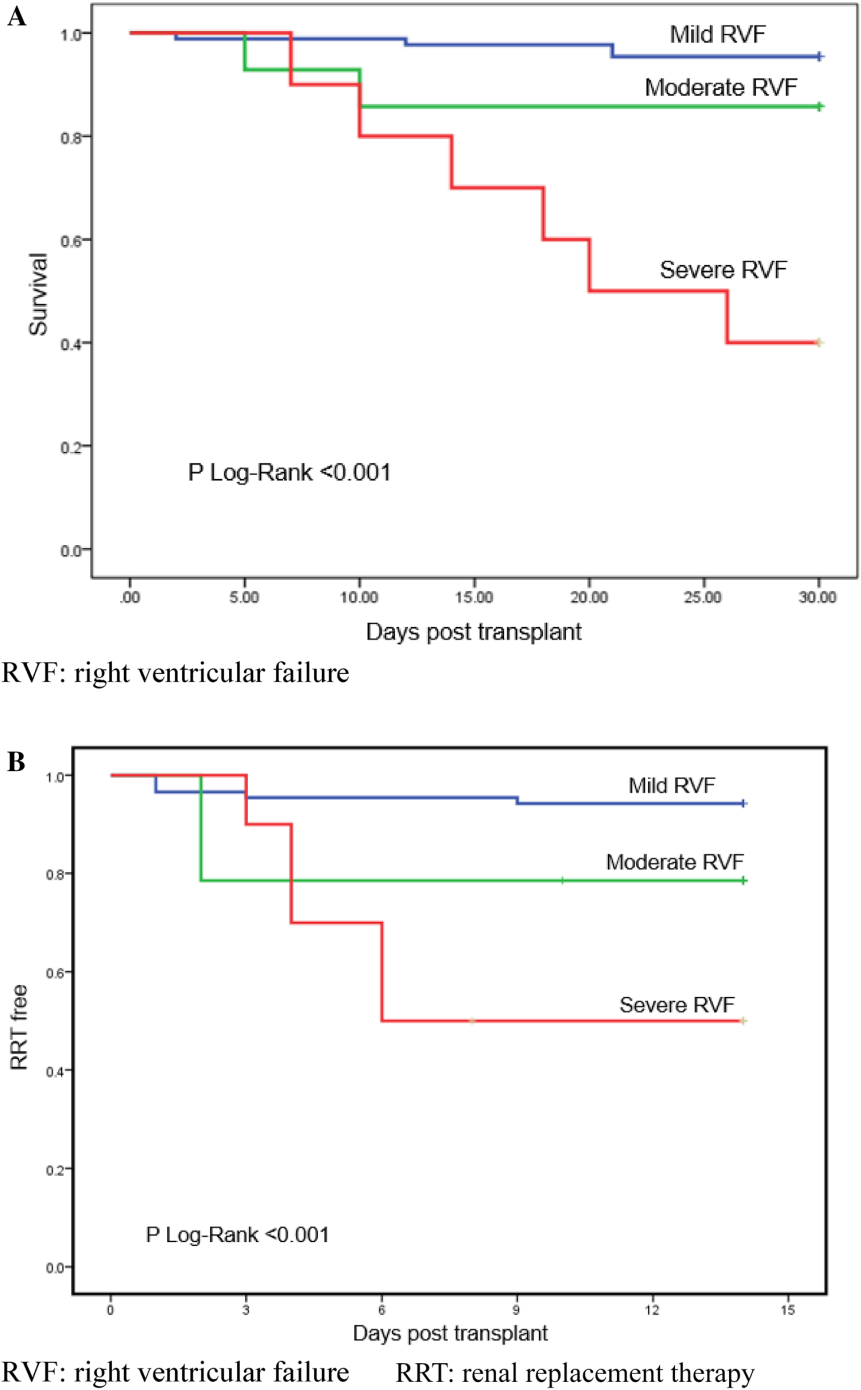
(**A**) Kaplan–Meier of 30-day survival in patients with primary isolated mild, moderate, severe right ventricular failure (RV) post-transplant. (**B**) Kaplan–Meier of dialysis-free survival curves in patients with mild, moderate, severe right ventricular failure post-transplant.

At 90 days post transplantation, no graft loss or ACR ≥ grade 2R was detected. By Kaplan–Meier analysis, severe RV failure was significantly associated with 30-day mortality and post-transplant dialysis (*p* Log-rank < 0.001 for both) (Fig. 4A,B). By univariate analysis, severe PI-RVF was a predictor of 30-day mortality and post-transplant dialysis (hazard ratio of 14.0, 95% CI 1.58–124.53%, *p* = 0.018 for 30-day mortality and hazard ratio of 22.8, 95% CI 2.02–257.40%, *p* = 0.011 for post-transplant dialysis) (Table 5). Among pre-transplant variables, univariate analysis showed that pre-operative elevated BUN and pre-operative elevated PVR were significantly associated with post-transplant dialysis and pre-operative elevated BUN and pre-operative elevated bilirubin were associated with 30-day mortality. Multivariate analysis showed that female recipients and pre-transplant direct bilirubin > 1.5 mg/dl were significant pre-operative predictors for post-transplant severe isolated RV failure (Table 6). There were no significant differences in overall death, 30-day mortality, and post-operative RRT among patterns (A, B, or C) of RV failure.

**Table 5:** Univariate analyses for predictors of 30-day mortality and post-transplant dialysis (RRT) in the absence of sepsis and CNI.

	RRT		30-day mortality	
	Univariate analysis		Univariate analysis	
	HR	*p*	HR	*p*
Age	1.02	0.406	1.01	0.620
Female	0.73	0.636	1.83	0.302
Pre-operative BUN > 43 mg/dl	6.38	0.001*	5.24	0.004*
Direct bilirubin > 1.5 mg/dl	2.06	0.205	3.48	0.031*
Pre-transplant PVR (Wood units)	1.25	0.027*	1.24	0.057
Pre-transplant INTERMAC 1–3	1.58	0.448	2.49	0.120
Donor age	0.98	0.479	0.99	0.789
Donor’s inotropic score	0.93	0.052	0.97	0.222
Ischemic time	1.00	0.606	0.99	0.255
Severe RV failure	6.90	0.001*	13.223	< 0.001*

*BUN* blood urea nitrogen, *INTERMACS* Interagency Registry for Mechanically Assisted Circulatory Support, *PVR* pulmonary vascular resistance, *RRT* renal replacement therapy, *RV* right ventricular.

*P value < 0.05 is statistical significance.

**Table 6:** Univariate and multivariate analyses for post-operative severe RV failure.

Variables	Severe RV failure			
	Univariate analysis		Multivariate analysis	
	OR	*p*	OR	*p*
Age	1.01	0.553	0.98	0.475
Female	2.74	0.133	10.8	0.047*
Pre-operative BUN > 43 mg/dl	10.15	0.001*	4.55	0.114
Direct bilirubin > 1.5 mg/dl	18.22	< 0.001*	8.9	0.018*
Pre-transplant PVR (Wood units)	1.02	0.922	1.04	0.870
Pre-transplant INTERMAC 1–3	0.80	0.789		
Donor age	1.03	0.444	1.01	0.835
Donor’s inotropic score	0.98	0.426		
Ischemic time	1.00	0.932	0.99	0.867

*BUN* blood urea nitrogen, *INTERMACS* Interagency Registry for Mechanically Assisted Circulatory Support, *PVR* pulmonary vascular resistance, *RRT* renal replacement therapy, *RV* right ventricular.

*P value < 0.05 is statistical significance.

## Discussion

**Figure 5:**
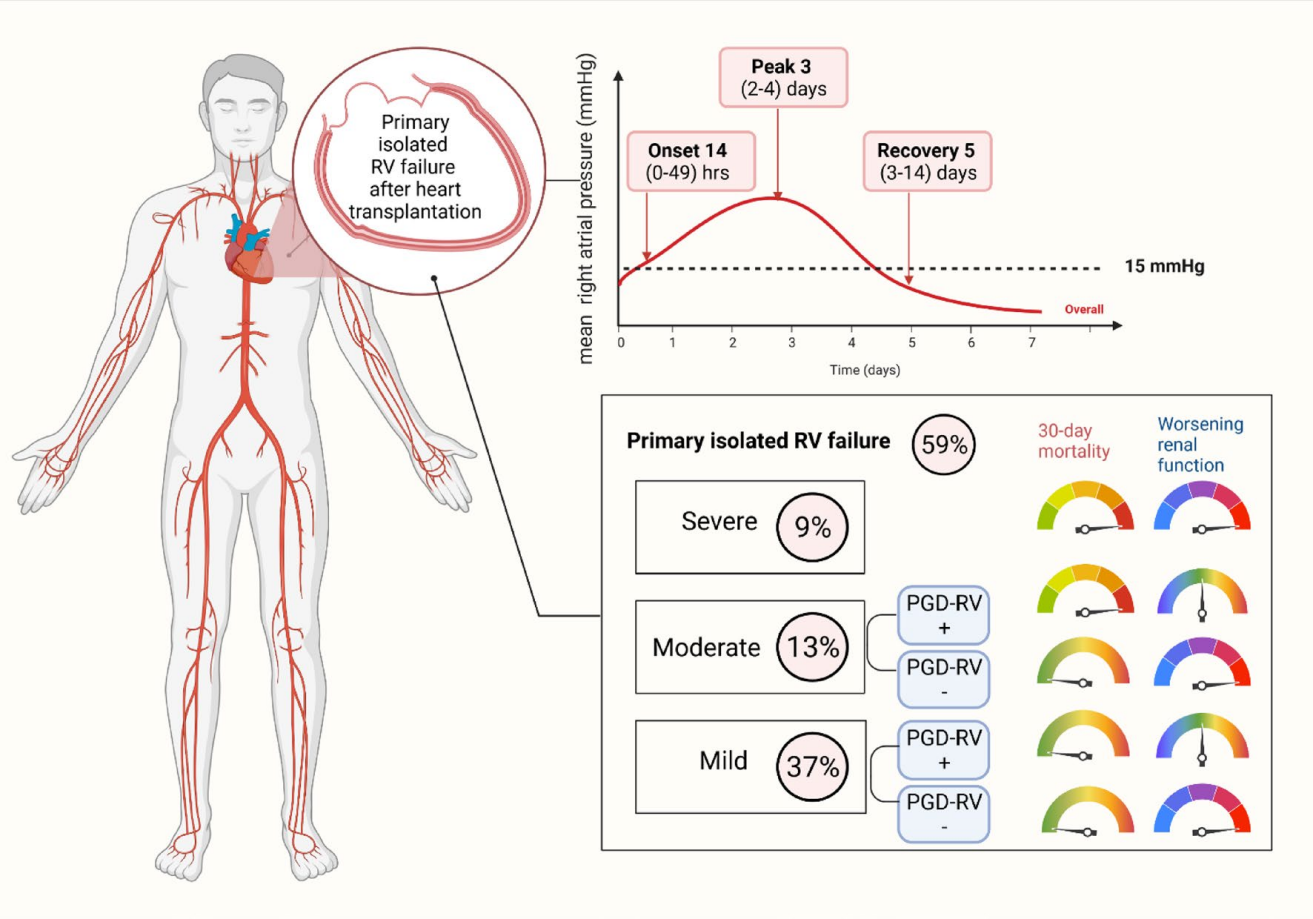
Central Illustration.

In this first reported transplant cohort from Southeast Asia, post-transplant PI-RVF is prevalent and mostly occurred during the first 24 h after completion of cardiac transplant surgery. However, delayed time to onset of RV failure after 24 h post-transplantation occurred in 45% of these patients precluding these cases to be defined as PGD-RV. The peak of RV failure (RAP) achieved its highest RAP values in day 3 after transplant surgery. RV failure transiently persisted for 4 days among all patients and 6 days in the subgroup of severe RV failure requiring MCS support. Excluding patients with severe PI-RVF or severe PGD-RV, only half of patients with mild to moderate RV failure met the criteria of mild to moderate PGD-RV due to the lack of evidence of early onset RV failure, CI < 2.0 L/min/m^2^, and PCWP < 15 mmHg. In the patients who did not meet the criteria of mild to moderate PGD-RV, the effect of RV failure on renal function was evident in about one-third of this subgroup. Severe RV failure was an independent predictor of 30-day post-transplant mortality and post-operative dialysis. Figure 5 represent central illustration of key findings.

### The prevalence of post-transplant primary isolated RV failure

In the present study, the prevalence of overall PI-RVF and *severe* PI-RVF requiring MCS (severe PGD-RV) were 59% and 9%, respectively. The prevalence of *severe* PI-RVF was similar to the prevalence of *severe* PGD-RV. Nicoara A. et al.^7^ reported*severe* PGD-RV present in 7% of post-transplant patients. However, PI-RVF after heart transplant surgery was more prevalent than PGD-RV. Sabatino et al.^5^ reported that PGD-RV was found in 17% of patients undergoing heart transplantation. The higher prevalence of PI-RVF in our study could be due to prolonged ischemic time (> 240 min) and a broader definition of PI-RVF..

### Post-transplant acute isolated RV failure vs. PGD-RV

We found that about half of patients with *mild* and *moderat*e PI-RVF post heart transplantation did not meet the criteria of *mild* and *moderate* PGD-RV mostly because time to onset of RAP > 15 mmHg occurred after 24 h post-cardiac transplant surgery. In patients with *severe* and *moderate* PI-RVF, about 20% had delayed time to onset of RV failure beyond 24 h post heart transplantation. The lack of a well-defined time point after completion of the cardiac transplant surgery may influence the onset time of elevated RAP. We proposed that the onset of a significant rise in RAP should expand beyond 24 h after completion of the cardiac transplant surgery or after completion of surgical chest closure as two patients requiring MCS developed the onset of a rise in RAP at 38- and 41-h post-surgery. Additionally, another 2 patients were precluded from PGD-RV because PCWP was 17 mmHg. Both patients had severely dilated RV and small LV on echocardiography. RV dilatation and interventricular septal shift to the left that leads to a smaller LV and increased left ventricular end-diastolic pressure in an intact pericardium have been described^17^. Diastolic ventricular interdependence can occur with or without an intact pericardium^18–22^. This was confirmed by an experimental study by Laster SB. et al.^23^ who demonstrated that RV dilatation and reverse septal curvature elevated left atrial pressure immediately after ligation of right coronary artery in closed-chest dogs. We proposed the cut-off for PCWP < 18 mmHg for diagnosis of PI-RVF as the shift in interventricular septal configuration in response to RV dilatation can cause mildly elevated LV filling pressure. We agreed with a simplified criteria using the escalation of inotropic requirement or inability to wean inotrope to define moderate PGD-RV proposed by Alam A. et al.^14^ as there was a limitation of achieving CI < 2 L/min/m^2^ in the condition of multiple inotrope supports post-transplantation. Using the original criteria of PGD-RV, the prevalence of PI-RVF post-transplant or PGD-RV is likely underestimated.

### Characteristics and mechanisms of post-transplant primary isolated RV failure

The right ventricular free wall is anatomically thin and vulnerable to ischemia^12^. The pathogenesis of post-heart transplant RV failure in the absence of pulmonary hypertension are multifactorial and complex^10,13^. The postulated mechanisms of RV failure include acute ischemic-reperfusion injury with RV myocardial stunning as a consequence of the rapid surge of catecholamines and multiple cytokines after brain death, cardiac myocyte damage caused by inotropes, the alteration of cellular metabolism of cardiac myocyte during cardiac preservation in a cold storage, and reperfusion injury after completion of surgical anastomosis^9,11,23–26^. The prolonged cold ischemic time is related to altered cellular metabolism and disturbance in Na + /K + ATPase pump resulting in cellular swelling and myocardial dysfunction^25,27^. We hypothesized that cellular swelling with myocardial stunning was the key mechanism of PI-RVF in our cohort as cold ischemic time was prolonged compared with other previous studies^5–7,13^. In our study, the median time to onset of elevated RAP of 15 mmHg or greater was 14 h after completion of surgery and reached its highest peak value of RAP at post-operative day 3. Consistently, Sicard et al^28^ confirmed RV dysfunction in mice model 24–48 h post-right coronary ligation. These findings also underscored mechanistic insight of RV failure in response to RV ischemic injury. We observed that RV failure in our study were transient and persisted for 4 (median) days among all patients and 6 days in the MCS group. Similarly, Laster et al.^23^ previously demonstrated that RV functional recovery of greater than 50% of baseline was evident at 4 days after coronary occlusion in the closed-chest dogs. Additionally, recent studies of MCS support for RV failure or PGD showed that the median duration of MCS support, suggestive of RV recovery, was 6–7 days^5,7^. Identifying patients who are at risk and timely MCS implantation are critically vital. We found that pre-operative elevated bilirubin and being female were predictors of severe PI-RVF requiring MCS post-transplant. Our findings align with the previous studies that have shown that higher serum bilirubin and female recipient were significant predictors of severe PGD -LV and/or RV^5,7,29^.

### Outcomes

Post-heart transplant severe RV dysfunction and severe PGD-RV are the leading cause of 30-day mortality post-transplantation^30,31^. We found that severe PI-RVF or severe PGD-RV was associated not only with 30-day mortality, but also with post-operative dialysis. Although mortality in moderate RV failure patients was lower than the severe PGD-RV group, worsening renal function and requirement of post-operative dialysis were still clinically significant. In our study, 2 of 7 patients (29%) with moderate RV failure who did not meet the PGD criteria also required post-operative dialysis in the absence of sepsis and contamination of CNI. Mean post-operative BUN and creatinine in patients with *moderate* PI-RVF was also higher than those with *mild* RV failure. These findings underscore that moderate PIVF may affect hemodynamics resulting in worsening renal function. Clinical significance of moderate RV failure should not be underestimated.

### Study limitations

The study is limited by its retrospective nature where bias may have influenced post-transplant management strategy and clinical outcomes. The prevalence and clinical outcomes were also affected by post-transplant care protocols, donor heart preservation techniques, surgical techniques, and geographic donor allocation associated with ischemic time in a single tertiary center which may limit generalizable. The 14-year period also introduces the potential for changes in treatment delivery as technology and skill may improve and protocols execution may evolve. These factors including improved surgical skills leading to shorter ischemic time, improved organ preservation techniques minimizing RV myocyte injury, and meticulous donor selection and management ultimately affect the prevalence of RV failure. The small number of patients in each subgroup of severity of RV failure precluded additional multivariate analyses. Further studies utilizing larger patient population may help to identify predictors across the spectrum of RV failure severity. The majority of MCS supports for severe PI-RVF in our study were ECMOs. Although RAP can guide volume management in ECMO supported patients, the RAP in the light of ECMO varies and depends on the position of venous cannula and flow pressure in the right atrium, in addition to RV function per se. The RV characteristics and RV recovery in patients with severe RV failure were limited to assessment of patients who were on MCS and died on MCS. We excluded patients with continuation of ECMO use from pre-transplant period because determination of the onset of elevated RAP post-transplant may not be accurate in those with mild or mild to moderate PI-RVF in the presence of ECMO. Additionally, pulmonary pressure was limited to assess in those patients. Lastly, the measurement of post-operative hemodynamic parameters was not validated for intra- and inter-observer variation These parameters were recorded by intensive care unit nurses as per clinically indicated protocol and were subjected to vary.

## Conclusions

Primary isolated RV failure was common after heart transplantation and characterized by transient and progressive RV functional deterioration occurring immediately or after 24 h post-transplant. The mean time to the highest RAP/peak RV failure was observed on post-operative day 3. The recovery of RV failure mostly occurred within 7 days after transplantation. These findings may represent transient and progressive swelling and stunning of RV myocardium as a consequence of ischemic-reperfusion injury in the first few days after heart transplantation. Despite this transient phenomenon, life threatening RV failure or severe RV failure requiring MCS occurred in about 10% of all cardiac transplantations and was associated with 30-day mortality and a need for dialysis post-transplant, while moderate isolated RV failure was associated with post-transplant worsening renal function and requirement of dialysis. A revised definition of PGD-RV to capture more patients with significant RV failure may be needed since those patients in our study who had adverse outcomes did not meet the criteria of PGD.

## Data Availability

The datasets generated and/or analyzed during the current study are not publicly available due to protecting the participants’ anonymity but are available from the corresponding author on reasonable request.
